# Broadening the Scope…

**DOI:** 10.1096/fba.2020-00007

**Published:** 2020-05-09

**Authors:** Ralph A. Bradshaw

**Affiliations:** ^1^ University of California Irvine CA USA

*FASEB BioAdvances* (*FBA*) has now moved into its second year of publication and appropriately it is planning to institute some new features to mark this event. The last year has seen some significant changes in the editorial team including the addition of four new Associate Editors and a near doubling of the editorial board. The journal has also expanded its outreach to potential authors through various efforts designed to increase its recognition and visibility and, in this regard, is planning several “special” issues that will be devoted to a single topic, which cover reversible acetylation, exosome structure and homeostasis, cancer neuroscience, and other areas.

Being mindful that we are a FASEB sponsored journal, we are also taking additional steps to reflect and enhance that relationship. In the first place we continue to add board members that will reflect the scientific interests of our member societies and bring a broader scope to the science we publish. To some degree, this is going to be an ongoing effort as we anticipate that FASEB will continue to grow in the months and years to come. In a recent decision, the FASEB Board of Directors voted to expand membership in the organization by creating an Associate Member status. In due course, it is anticipated that there will be growth in both the regular and associate categories and *FBA* will strive to serve all in the content that it publishes. More detailed information regarding FASEB membership categories can be found at https://membership.FASEB.org

In addition to the research interests of the scientists that make up the membership rosters of its constituent societies, FASEB has several programs that provide support to and advocacy for biomedical research funding. Indeed, the Office of Public Affairs (OPA) at FASEB is a mainstay of these efforts and is widely regarded as the voice of bioscientists in the policy arena in Washington and beyond. *FBA* is pleased to announce that Dr. Howard Garrison, who headed OPA for 25 years before retiring in 2018, has joined our editorial team as a Special Editor for Policy. He will initiate a program to publish scholarly contributions that describe, analyze, and discuss issues that impact the conduct and regulation of biomedical research and its practitioners. In addition, Dr. Garrison, with two colleagues, has recently completed an authoritative history of FASEB covering 105 years (1912‐2017) entitled *The Federation of American Societies of Experimental Biology: A Century of Service and Advocacy* (which is available through FASEB; information at https://faseb.directfrompublisher.com/). His mandate as an *FBA* Special Editor will also cover the history of bioscience. As a part of this latter section, *FBA* will begin publishing obituaries of notable individuals in the bioscience community in an *In Memoriam* subsection. We expect initial contributions to both categories shortly.

Biomedical education is a third area that FASEB has strong interests in and *FBA* plans to begin immediately the publication of articles dealing with the challenges of this area. Dr. Michael Hortsch, an Associate Editor of *FBA,* has agreed to lend his considerable expertise to this endeavor and will act as a Special Editor for Education in addition to his regular duties as an AE. The first article in what we plan as an extended series in the educational area appears in the current issue. In due course we expect to have one or more special issues devoted to educational matters to compliment the special issues on science topics noted above.

The motives behind these expansions in scope are twofold: first, we are desirous that *FBA* better serves the member societies of FASEB by devoting portions of the journal to articles that deal with core FASEB values and interests; and second, while we are proud of our relationship with our sister journal, *FASEB Journal*, we are keen to establish our own identity. By broadening the scope of our content, we hope to accomplish both goals. We await the reactions of our readership, and any comments, suggestions and concerns will be welcome. We are a very young publication, which has its advantages and disadvantages, but one of the former is the ability to make innovative changes quickly and efficiently.

